# Transcriptome and Small RNA Combined Sequencing Analysis of Cold Tolerance in Non-heading Chinese Cabbage

**DOI:** 10.3389/fgene.2021.605292

**Published:** 2021-07-21

**Authors:** Jin Wang, Qinxue Zhang, Xiong You, Xilin Hou

**Affiliations:** ^1^State Key Laboratory of Crop Genetics and Germplasm Enhancement/Key Laboratory of Biology and Germplasm Enhancement of Horticultural Crops in East China, Ministry of Agriculture/Engineering Research Center of Germplasm Enhancement and Utilization of Horticultural Crops, Ministry of Education, Nanjing Agricultural University, Nanjing, China; ^2^School of Life Sciences, Jiangsu University, Zhenjiang, China; ^3^College of Sciences, Nanjing Agricultural University, Nanjing, China

**Keywords:** non-heading Chinese cabbage, transcriptome sequencing, small RNA sequencing, cold tolerance, expression pattern

## Abstract

**Background:**

Non-heading Chinese cabbage (*Brassica rapa* ssp. *chinensis*) is an important leaf vegetable grown worldwide. However, there has currently been not enough transcriptome and small RNA combined sequencing analysis of cold tolerance, which hinders further functional genomics research.

**Results:**

In this study, 63.43 Gb of clean data was obtained from the transcriptome analysis. The clean data of each sample reached 6.99 Gb, and the basic percentage of Q30 was 93.68% and above. The clean reads of each sample were sequence aligned with the designated reference genome (*Brassica rapa, IVFCAASv1*), and the efficiency of the alignment varied from 81.54 to 87.24%. According to the comparison results, 1,860 new genes were discovered in Pak-choi, of which 1,613 were functionally annotated. Among them, 13 common differentially expressed genes were detected in all materials, including seven upregulated and six downregulated. At the same time, we used quantitative real-time PCR to confirm the changes of these gene expression levels. In addition, we sequenced miRNA of the same material. Our findings revealed a total of 34,182,333 small RNA reads, 88,604,604 kinds of small RNAs, among which the most common size was 24 nt. In all materials, the number of common differential miRNAs is eight. According to the corresponding relationship between miRNA and its target genes, we carried out Gene Ontology and Kyoto Encyclopedia of Genes and Genomes enrichment analysis on the set of target genes on each group of differentially expressed miRNAs. Through the analysis, it is found that the distributions of candidate target genes in different materials are different. We not only used transcriptome sequencing and small RNA sequencing but also used experiments to prove the expression levels of differentially expressed genes that were obtained by sequencing. Sequencing combined with experiments proved the mechanism of some differential gene expression levels after low-temperature treatment.

**Conclusion:**

In all, this study provides a resource for genetic and genomic research under abiotic stress in Pak-choi.

## Background

Non-heading Chinese cabbage (*Brassica rapa* ssp. *chinensis*), also known as Pak-choi, is one of the most common cultivated vegetables in China. Its growth and productivity are often adversely affected by cold and even freezing stresses from the environment (Abe et al., 2003; Yu et al., 2010; Li et al., 2016). The response mechanism of *Arabidopsis*’ cold acclimation and freezing resistance is not exactly the same (Rahman et al., 2020). The cold domestication of plants, which usually involves many biochemical and physiological changes, is complicated and difficult to understand (Salinas, 2002; Kaplan et al., 2004; Chinnusamy et al., 2007; Krasensky and Jonak, 2012). In *Arabidopsis*, the latest study found that the cold stress response was mediated by the GNOM ARF-GEF pathway (Ashraf and Rahman, 2019). After low-temperature treatment, the metabolism and transcriptome of plants are greatly affected, and the expression levels of certain genes are also regulated. Some related metabolic enzymes are inhibited, and the degree of plant metabolism are affected to some extent (Chinnusamy et al., 2007). Nowadays, genetic, biochemical, and various sequencing methods have been applied to detect how some genes cope with environmental stresses (Chinnusamy et al., 2007; Krasensky and Jonak, 2012), and many plants have been studied today, such as rice (Hussain et al., 2016; Wang et al., 2016), cotton (Zhao et al., 2012), tomato (Starck et al., 2000), potato (Shinozaki and Yamaguchi-Shinozaki, 1996), muskmelons (Wang et al., 2004), and sugarcane (Thakur et al., 2010; Anjum et al., 2011; Zhu et al., 2013). However, the response mechanism of non-heading Chinese cabbage to cold stress remains unclear, and further joint research is needed.

Nowadays, transcriptome analysis has gradually become a useful and general tool for discovering genes in multiple stress pathways, including determining the expression patterns of related genes (Martin and Wang, 2011; Hamilton and Robin Buell, 2012; Ward et al., 2012; Li et al., 2020). The related genes reported in plant secondary metabolism were discovered based on experimental methods of functional genome sequencing (Dixon, 2001; Goossens et al., 2003). In addition, RNA sequencing technology is often used to obtain complete transcriptome information from different plants, such as tea tree, chlorophytum borivilianum, and atractylodes lancea, and provide better insights into transcription or posttranscriptional force, including regulation of essential genes, during secondary metabolite biosynthetic pathways (Kalra et al., 2013; Li et al., 2015; Devi et al., 2016). Nowadays, transcriptome sequencing has been successfully used to detect expression levels of related genes in many organisms, such as rice (Zhang et al., 2010), yeast (Nagalakshmi et al., 2008; Wilhelm et al., 2008), sweet potato (Wang et al., 2010), and taxus (Ge et al., 2011). With Illumina sequencing technology, millions of sequences were read at a time, and individual assembled genes were mapped into a reference transcriptome map for molecular annotation (Cheng et al., 2015).

Studies have found that microRNA (miRNA), trans short interfering RNA (ta-siRNA), and heterochromatic short interfering RNA (hc-siRNA), all play important roles in different organisms (Axtell, 2013). Among them, miRNA, a type of endogenous small RNA, is composed of about 22 nucleotides (nt) and usually plays a negative role in regulating gene expression (Voinnet, 2009). Many studies have shown that miRNAs are often involved in plant development, hormone signaling, and abiotic stress responses (Jones-Rhoades et al., 2006; Chambers and Shuai, 2009). Generally speaking, small interfering RNAs are processed from perfect double-stranded RNA, while miRNAs are derived from single-stranded RNA transcripts, forming an imperfect double-stranded stem-loop precursor structure (Llave et al., 2002; Khraiwesh et al., 2010; Hao et al., 2012; Szittya and Burgyán, 2013). On the whole, miRNA plays a vital role in various biological and metabolic processes of plant growth and development, such as biotic (or abiotic) stresses, which can also negatively regulate the expression of target genes, by inhibiting (cutting) target mRNA or other ways (Bartel, 2004; Jones-Rhoades and Bartel, 2004; Jones-Rhoades et al., 2006; Mallory and Vaucheret, 2006; Sunkar et al., 2006; Voinnet, 2009; Wu et al., 2010). Therefore, it is important to identify miRNAs and their target genes (or miRNAs), which is essential for a better understanding of miRNA-mediated regulation of cold stress genes.

In this study, we compared the tolerance of two common and typical non-heading Chinese cabbage varieties, *Suzhouqing* (*BcL.1*) and *Sijiucaixin* (*BcL.2*), to cold stress. We found that *BcL.1* is more tolerant to cold stress compared with *BcL.2*. We used RNA-Seq for comprehensive characterization and explored the effects of low temperature. We identified the most important genes in the low-temperature response and discussed their regulatory networks under cold stress. Furthermore, we identified conserved and novel miRNAs and their potential target genes in non-heading Chinese cabbage, and discussed the possible connections between them. Quantitative real-time polymerase chain reaction (qRT-PCR) was also used to assess the expression levels of common differentially expressed genes (DEGs) and identify those candidate genes involved in cold tolerance. This work might serve as a reference of the functional analysis of cold tolerance in non-heading Chinese cabbage.

## Results

### Quantity Statistics and Venn Diagram of Differentially Expressed Genes

**FIGURE 1:**
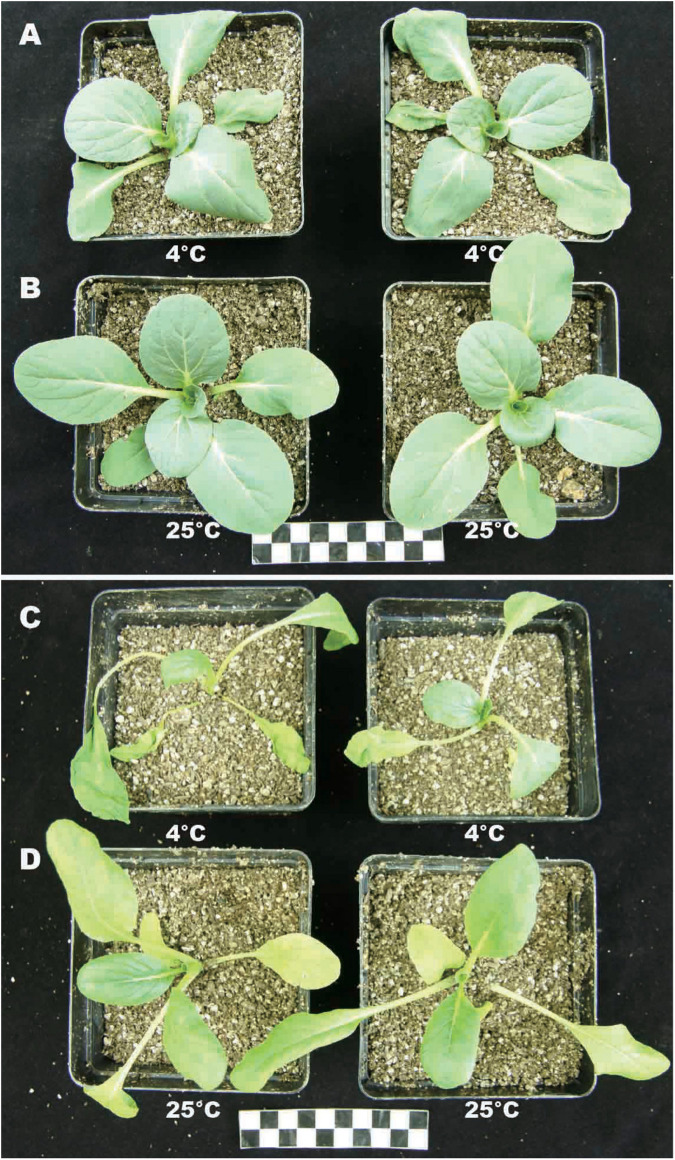
Phenotype of *BcL.1* and *BcL.2* under cold stress. Approximately 30-day-old seedlings were subjected to cold stress (4°C for 6 h); plants grown under normal condition (22°C) for 6 h were used as control. **(A)** *BcL.1-4*. **(B)** *BcL.1-25*. **(C)** *BcL.2-4*. **(D)** *BcL.2-25*.

To study the effects of temperature on plant growth, plants (*BcL.1* and *BcL.2*) were grown for 6 h in environments of 25 and 4°C. Except for the temperature, the other conditions remain unvaried. Then, we observed that low temperatures have an important effect on plant phenotype. Whether it is *BcL*.*1* or *BcL*.*2*, under 4°C treatment, plant leaves are more likely to shrink or even wither than under the 25°C treatment (Figure 1). This result corresponds to previous reports that cold stress usually downgrades the seedling vigor (Kang and Saltveit, 2002; Wang et al., 2016) and causes leaf atrophy, slows crop growth, and ultimately reduces the yield (Cruz and Milach, 2004; Oliver et al., 2007; Ruelland et al., 2009).

**FIGURE 2:**
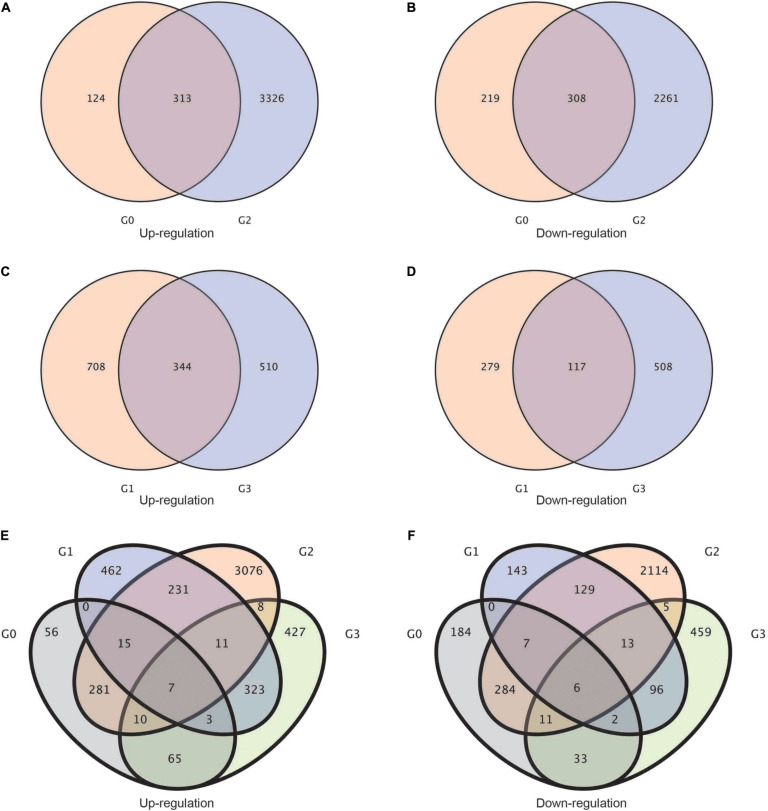
Statistics of the common genes and specific genes in different groups. **(A,B)** Venn diagram of up-/downregulated differentially expressed genes between G2 and G0 groups. **(C,D)** Venn diagram of up-/downregulated differentially expressed genes between G3 and G1 groups. **(E,F)** Venn diagram of up/downregulated differentially expressed genes in all groups. G0: *BcL.2-25* vs. *BcL.2-4*; G1: *BcL.1-25* vs. *BcL.2-25*; G2: *BcL.1-25* vs. *BcL.1-4*; G3: *BcL.1-4* vs. *BcL.2-4*.

Afterward, to study the upregulation and downregulation of common genes shared by each group of treatments, we established a Venn diagram of differentially expressed genes. Between the G0 (*BcL.2-25* vs. *BcL.2-4*) and G2 (*BcL.1-25* vs. *BcL.1-4*) groups, a total of 313 common genes were upregulated, and 308 common genes were downregulated (Figures 2A,B). Meanwhile, between the G1 (*BcL.1-25* vs. *BcL.2-25*) and G3 (*BcL.1-4* vs. *BcL.2-4*) groups, a total of 344 common genes were found to be upregulated, and 117 common genes were downregulated (Figures 2C,D). In all materials, a total of seven common genes were upregulated, and six common genes were downregulated (Figures 2E,F and Supplementary Table 1). We speculated that these DEGs might help increase the potential application value of non-heading Chinese cabbage under cold stress.

### Functional Annotation and Classification

**FIGURE 3:**
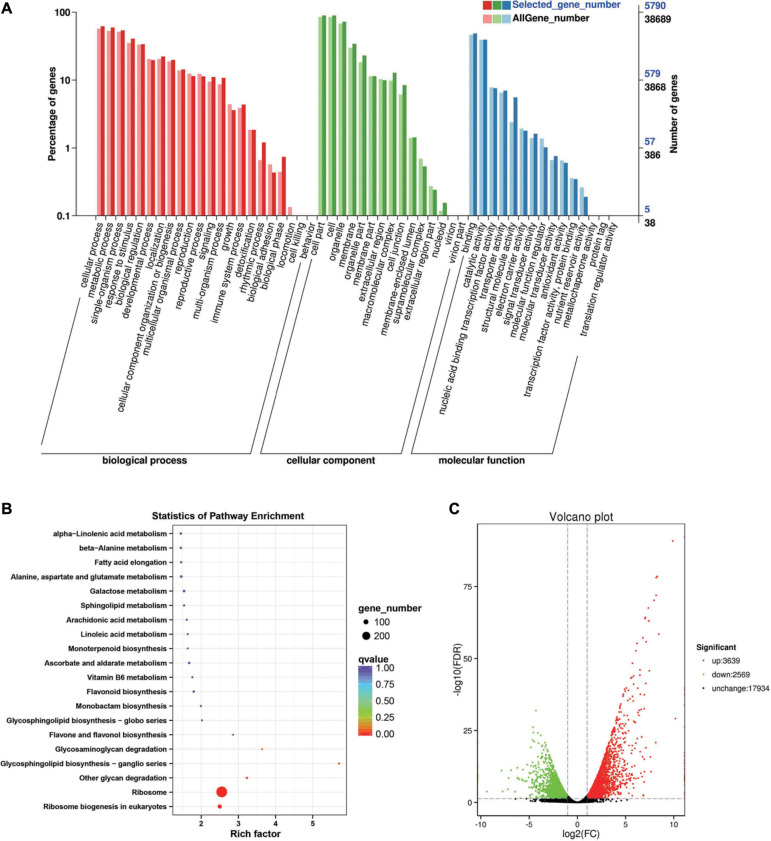
Enrichment analysis of differentially expressed genes between C2 and C1 groups. **(A)** Gene Ontology (GO) classification statistics of differentially expressed genes. It mainly includes three branches: biological process, cellular component, and molecular function. **(B)** Kyoto Encyclopedia of Genes and Genomes (KEGG) pathway enrichment statistics of differentially expressed genes. **(C)** Volcano plot statistics of the number of differentially expressed genes. C1: *BcL.1-25*, C2: *BcL.1-4*.

Between the *BcL.1-25* and *BcL.1-4* groups, 6,208 DEGs (*p* < 0.05) were detected, including 3,639 upregulated and 2,569 downregulated genes (Figure 3C). The annotated unigenes were then assigned to Gene Ontology (GO) terms for functional classification. Three main categories (biological process, molecular function, and cellular component) of GO classification were analyzed separately to investigate their functional distribution. To simplify the functional distribution of plants, we assigned the annotated sequences to GO-slim terms to obtain a “thin” version of classification (Gao et al., 2015). Cellular process (GO:0009987, 3,589 genes) and metabolic process (GO:0008152, 3,424 genes) in the biological process, cell part (GO:0004464, 5,170 genes) and cells (GO:0005623, 5,169 genes) in the cellular component and binding activity (GO:0005488, 2,808 genes), and catalytic activity (GO:0003824, 2,279 genes) in the molecular function were the most representative level 2 GO terms in all three data sets, respectively (Figure 3A and Supplementary Table 2). To further identify the active biochemical pathways, we mapped it to the reference canonical pathways in the Kyoto Encyclopedia of Genes and Genomes (KEGG). KEGG is thought to provide a basic platform for systematic analysis of gene function in terms of the network of gene products (Kanehisa and Goto, 2000). A total of 24,199 unigenes were annotated based on a BLASTX search of the KEGG database (Supplementary Table 3): 263 biosynthesis pathways were predicted and classified into five categories, of which the ribosome pathway was the largest, containing 287 genes (287 out of 1,586, 18.10%) (Figure 3B, Supplementary Figure 1A, and Supplementary Table 10). The annotated unigenes were categorized into different functional groups based on the Cluster of Orthologus Groups (COG) database (Supplementary Table 14). Unigenes (3,700) could be classified into 23 COG categories. Out of the 3,700 unigenes, general function prediction only (679, 18.35%) was assigned to the COG category of general function prediction, which represented the largest functional group of the 23 COG categories, followed by translation, ribosomal structure, and biogenesis (433, 11.70%), transcription (329, 8.90%), replication, recombination and repair (293, 7.92%), and signal transduction mechanisms (290, 7.84%) (Supplementary Figure 2A).

**FIGURE 4:**
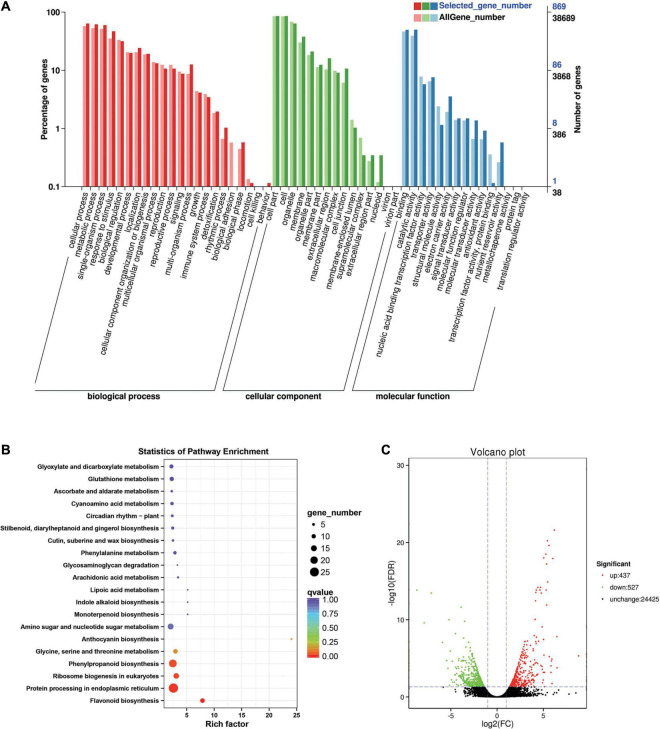
Enrichment analysis of differentially expressed genes between C4 and C3 groups. **(A)** GO classification statistics of differentially expressed genes. It mainly includes three branches: biological process, cellular component, and molecular function. **(B)** The KEGG pathway enrichment statistics of differentially expressed genes. **(C)** Volcano plot statistics of the number of differentially expressed genes. C3: *BcL.2-25*, C4: *BcL.2-4*.

Between the *BcL.2-25* and *BcL.2-4* groups, 964 DEGs (*p* < 0.05) were detected, including 437 upregulated and 527 downregulated genes (Figure 4C). Through GO enrichment stratification analysis, cellular process (GO:0009987, 552 genes) and metabolic process (GO:0008152, 534 genes) in the biological process, cell part (GO:0004464, 741 genes) and cells (GO:0005623, 741 genes) in the cellular component and binding activity (GO:0005488, 429 genes), and catalytic activity (GO:0003824, 433 genes) in the molecular function were the most representative level 2 GO terms, respectively (Figure 4A and Supplementary Table 4). Through KEGG pathway enrichment analysis (Supplementary Table 5), the protein processing in the endoplasmic reticulum pathway was the largest, containing 26 genes (26 out of 251, 10.36%) (Figure 4B, Supplementary Figure 1B, and Supplementary Table 11). Through COG classification of differentially expressed genes, out of 554 unigenes (Supplementary Table 15), general function prediction only (109, 19.68%) was assigned to the COG category of general function prediction, which represented the largest functional group of the 21 COG categories, followed by posttranslational modification, protein turnover, chaperones (59, 10.65%), amino acid transport and metabolism (49, 8.84%), carbohydrate transport and metabolism (45, 8.12%), and signal transduction mechanisms (35, 6.32%) (Supplementary Figure 2B).

**FIGURE 5:**
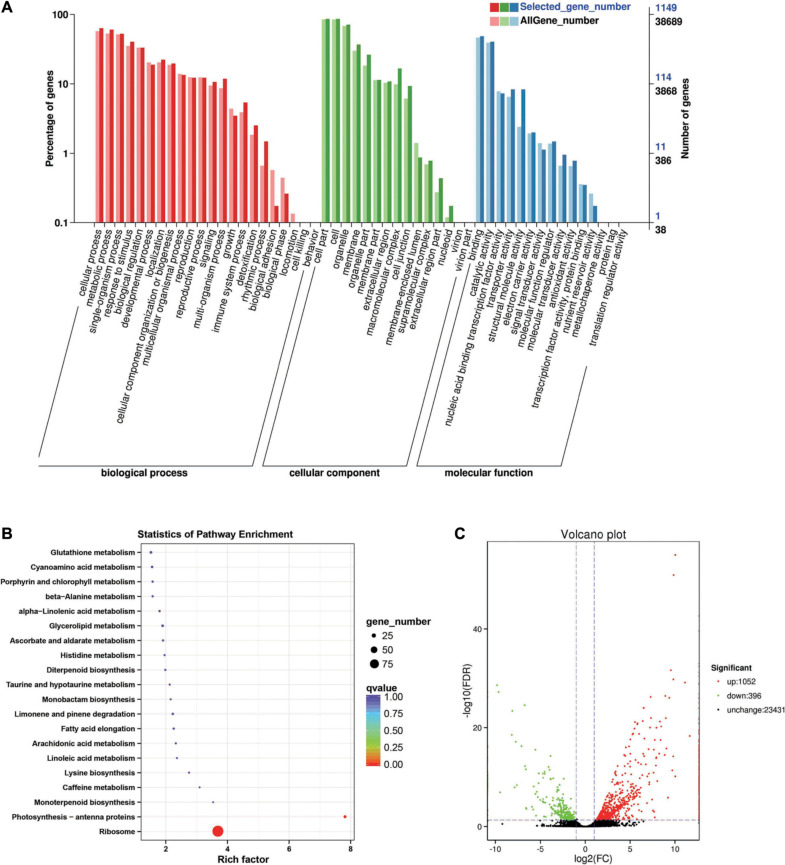
Enrichment analysis of differentially expressed genes between C3 and C1 groups. **(A)** GO classification statistics of differentially expressed genes. It mainly includes three branches: biological process, cellular component, and molecular function. **(B)** KEGG pathway enrichment statistics of differentially expressed genes. **(C)** Volcano plot statistics of the number of differentially expressed genes. C1: *BcL.1-25*, C3: *BcL.2-25*.

Between the *BcL.1-25* and *BcL.2-25* groups, 1,448 DEGs (*p* < 0.05) were detected, including 1,052 upregulated and 396 downregulated genes (Figure 5C). Through GO enrichment stratification analysis, cellular process (GO:0009987, 729 genes) and metabolic process (GO:0008152, 695 genes) in the biological process, cell part (GO:0004464, 988 genes) and cells (GO:0005623, 989 genes) in the cellular component and binding activity (GO:0005488, 556 genes), and catalytic activity (GO:0003824, 465 genes) in the molecular function were the most representative level 2 GO terms, respectively (Figure 5A and Supplementary Table 6). Through KEGG pathway enrichment analysis (Supplementary Table 7), the ribosome pathway was the largest, containing 96 genes (96 out of 366, 26.23%) (Figure 5B, Supplementary Figure 1C, and Supplementary Table 12). Through COG classification of differentially expressed genes, out of 681 unigenes (Supplementary Table 16), general function prediction only (124, 18.21%) was assigned to the COG category of general function prediction, which represented the largest functional group of the 23 COG categories, followed by translation, ribosomal structure, and biogenesis (109, 16.01%), transcription (51, 7.49%), replication, recombination, and repair (50, 7.34%), and amino acid transport and metabolism (44, 6.46%) (Supplementary Figure 2C).

**FIGURE 6:**
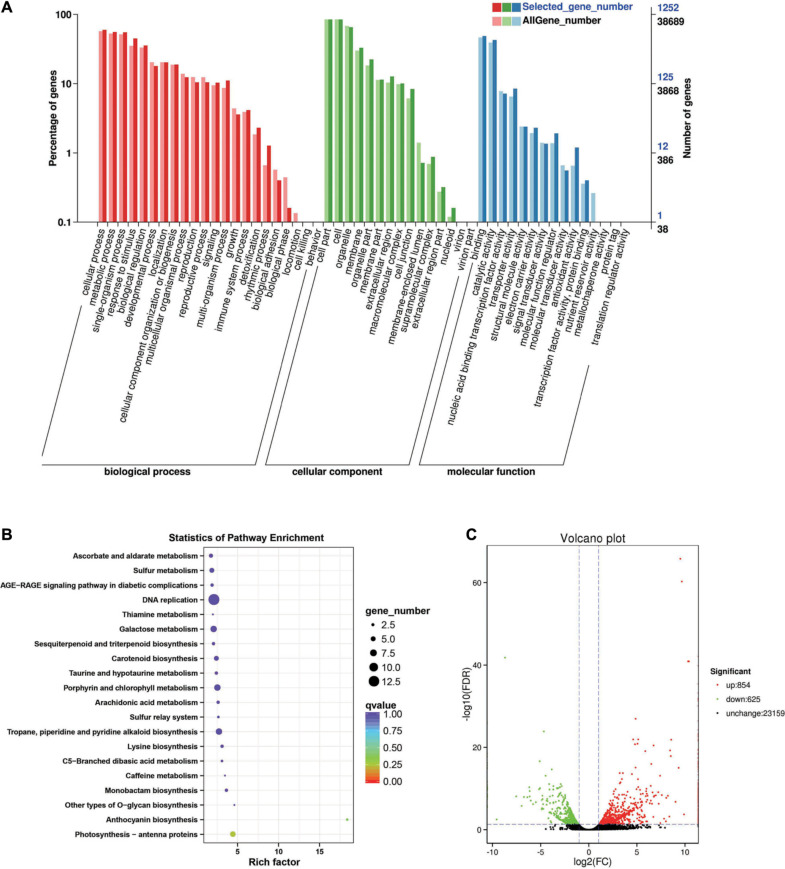
Enrichment analysis of differentially expressed genes between C4 and C2 groups. **(A)** GO classification statistics of differentially expressed genes. It mainly includes three branches: biological process, cellular component, and molecular function. **(B)** KEGG pathway enrichment statistics of differentially expressed genes. **(C)** Volcano plot statistics of the number of differentially expressed genes. C2: *BcL.1-4*, C4: *BcL.2-4*.

Between the *BcL.1-4* and *BcL.2-4* groups, 1,479 DEGs (*p* < 0.05) were detected, including 854 upregulated and 625 downregulated genes (Figure 6C). Through GO enrichment stratification analysis, cellular process (GO:0009987, 750 genes) and metabolic process (GO:0008152, 697 genes) in the biological process, cell part (GO:0004464, 1,056 genes) and cells (GO:0005623, 1,057 genes) in the cellular component and binding (GO:0005488, 610 genes), and catalytic activity (GO:0003824, 536 genes) in the molecular function were the most representative level 2 GO terms, respectively (Figure 6A and Supplementary Table 8). Through the KEGG pathway enrichment analysis (Supplementary Table S9), the DNA replication pathway was the largest, containing 13 genes (13 out of 329, 3.95%) (Figure 6B, Supplementary Figure 1D, and Supplementary Table 13). Through COG classification of differentially expressed genes, out of 681 unigenes (Supplementary Table 17), general function prediction only (144, 17.98%) was assigned into the COG category of general function prediction, which represented the largest functional group of the 23 COG categories, followed by carbohydrate transport and metabolism (74, 9.24%), posttranslational modification, protein turnover, chaperones (67, 8.36%), replication, recombination and repair (65, 8.11%), and amino acid transport and metabolism (58, 7.24%) (Supplementary Figure 2D).

### Clustering and Functional Enrichment of Differentially Expressed Genes in All Treatments

Among them, 13 DEGs were detected in all treatments, including seven upregulated and six downregulated. Some of the DEGs were involved in response to stress (GO:0006950) and stimulus (GO:0050896), as well as response to abiotic stress (GO:0009628), such as freezing (GO:0050826), cold (GO:0009409), and salt (GO:0009651) stress. Some of the DEG respond to growth hormone (GO:0060416) and water deprivation (GO:0009414) (Supplementary Table 1). In addition, we performed cluster analysis on all screened differentially expressed genes (Figures 3C, 4C, 5C, 6C). Nearly all differentially expressed genes are upregulated between *BcL.1-25* (N-25) and *BcL.1-4* (N-4) groups; between *BcL.2-25* (B-25) and *BcL.2-4* (B-4) groups, most of the genes were upregulated, while the *BcL.2-25* (B-25) group showed more upregulated genes; between *BcL.1-25* (N-25) and *BcL.2-25* (B-25) groups, the performance of upregulated genes and downregulated genes is very similar to that between *BcL.1-4* (N-4) and *BcL.2-4* (B-4) groups (Supplementary Figure 3).

### Quantitative Real-Time-Polymerase Chain Reaction Validation of the Candidate Differentially Expressed Genes Responsive to Cold Tolerance

**FIGURE 7:**
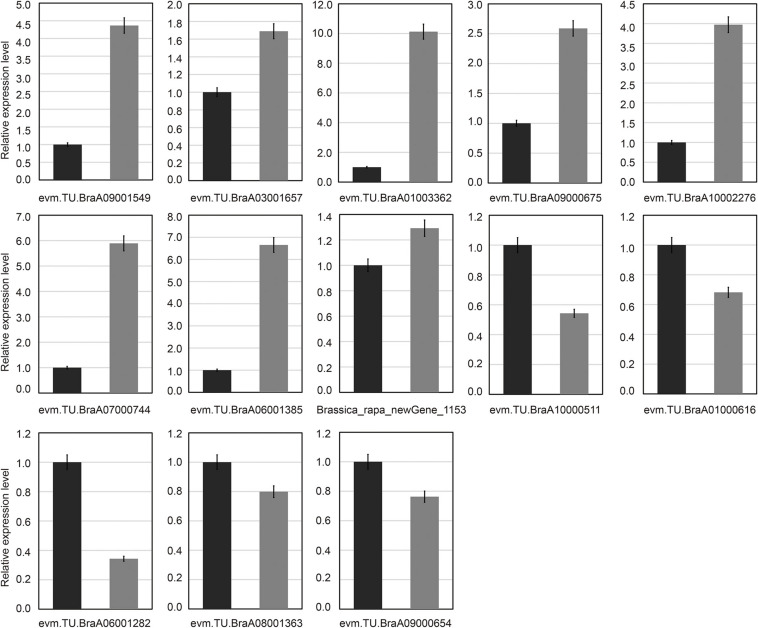
Verification of common differentially expressed genes by quantitative real-time polymerase chain reaction (qRT-PCR). Thirteen common differentially expressed genes (DEGs) were chosen for qRT-PCR validation. The relative expression levels of each gene were expressed as the fold change between *BcL.1-25* (black column) and *BcL.1-4* (gray column).

To test the reliability of the transcriptome sequencing results, qRT-PCR analysis was used. In this study, 13 common candidate DEGs were selected and detected in all treatments by qRT-PCR analysis (Figure 7). The results of transcriptome sequencing were compared with the results of qRT-PCR experiments. Our results showed that even if the fold changes in the expression levels of certain genes detected by transcriptome sequencing and qRT-PCR analysis did not match, almost all expression levels analyzed by qRT-PCR were highly consistent with the transcriptome sequencing results. These results also confirmed the reliability of transcriptome sequencing data (Figure 7). Through qRT-PCR analysis, it was found that there was only one downregulated gene (*Brassica*_*rapa*_new gene_1,153), and its expression level was different from the RNA-Seq data (Figure 7).

### Overview of Small RNA Sequencing Data

**FIGURE 8:**
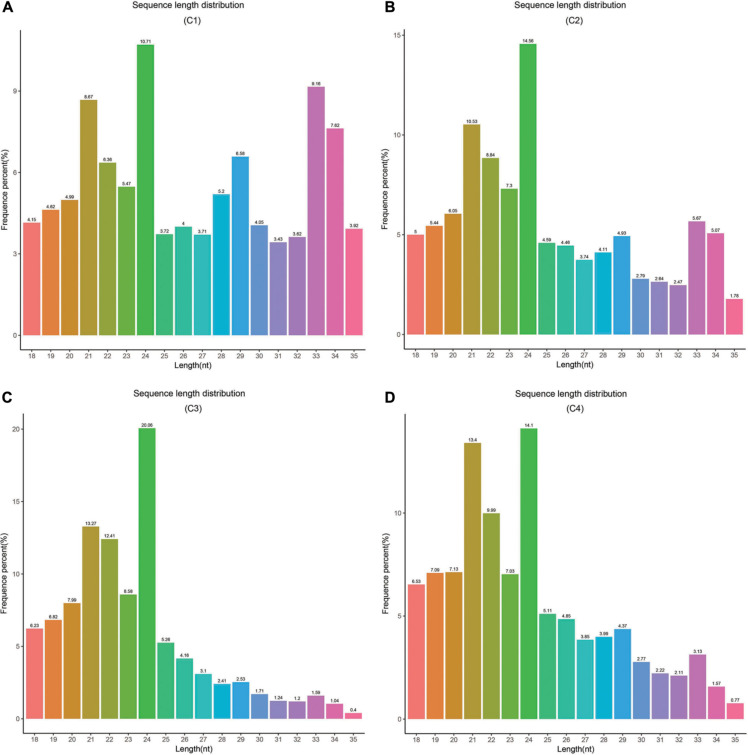
Length distribution and abundance of small RNAs in four libraries from Pak-choi. **(A)** Frequence percentage (%) and length of sRNA distribution in C1 (*BcL.1-25*) group. **(B)** Frequence percentage (%) and length of sRNA distribution in C2 (*BcL.1-4*) group. **(C)** Frequence percentage (%) and length of sRNA distribution in the C3 (*BcL.2-25*) group. **(D)** Frequence percentage (%) and length of sRNA distribution in the C4 (*BcL.2-4*) group.

In this study, these samples included C1 (*BcL.1-25*), C2 (*BcL.1-4*), C3 (*BcL.2-25*), and C4 (*BcL.2-4*), which were collected, sequenced, and analyzed. Total reads (34,182,333) were generated, and 8,836,042 unique reads were isolated. After removing low-quality reads, the length distribution of the small RNAs (18–35 nt) revealed that a length of 24 nt was the most abundant class among both clean and unique reads in all groups (Figure 8 and Table 1).

**TABLE 1:** Type and quantity of miRNA.

Sample	Total reads	Total bases (bp)	Uniq reads	Uniq bases (bp)
C1	7,404,323	195,732,796	1,708,990	42,044,898
C2	9,762,950	244,486,883	2,480,460	59,410,849
C3	8,345,217	194,120,302	2,669,504	62,223,537
C4	8,669,843	207,657,265	1,977,088	47,080,337

*Sample: The sample ID; Total reads: the total number of sRNAs; Total bases (bp): The total length of the sRNA; Uniq reads: the type of sRNA; Uniq bases (bp): The total length of various sRNAs.*

### Analysis of Known miRNA

**FIGURE 9:**
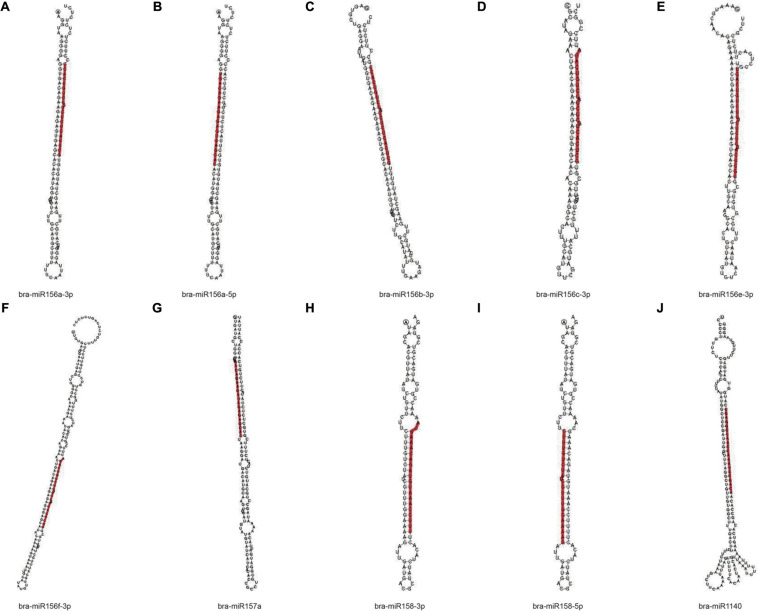
Secondary structure of the known miRNA on the match. **(A–J)** Secondary structure of 10 known miRNAs on the match from Pak-choi. The entire sequence is miRNA precursor, and the red highlight is where the mature sequence is located.

In order to obtain the details of the miRNA matched on each sample, the abovementioned reads were mapped to the reference sequence, which are compared with the specified range of sequences in miRBase, including the secondary structure of the known miRNAs on the match, and the information on the sequence, length, and number of occurrences of the miRNA in the present invention. When the miRNA developed into a mature body from the precursor, the process was completed by dicer digestion. The specificity of the cleavage site makes the miRNA mature sequence the first base. There was a strong bias, so the first base distribution of miRNAs of different lengths was also carried out, in addition to the base distribution statistics of the miRNAs. As shown in Table 2, the number of miRNAs in the C4 group was the highest, 338,668, and the number of miRNAs in the C1 group was the least, 129,932. However, in the C3 group, the types of miRNAs were the most, 1,774; the C1 group had the least types, 1,244. In Figure 9, we listed the secondary structure of the 10 known miRNAs on the match.

**TABLE 2:** Known miRNA alignment table for each sample.

Types	Total	C1	C2	C3	C4
Mapped mature	110	78	91	95	91
Mapped hairpin	80	73	75	77	77
Mapped uniq miRNA	6,168	1,244	1,692	1,774	1,458
Mapped total miRNA	868,276	129,932	196,191	203,485	338,668

### Predicted New miRNA

**FIGURE 10:**
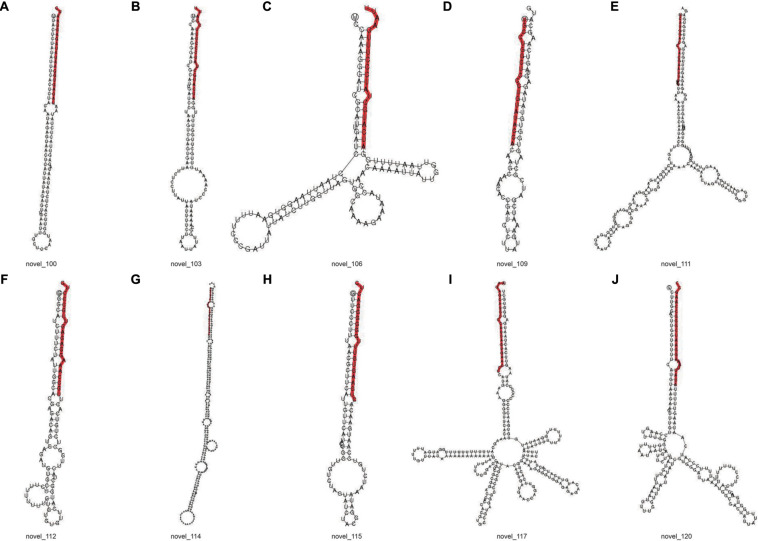
Secondary structure of predicted new miRNA. **(A–J)** Secondary structure of 10 predicted new miRNA. The entire sequence is miRNA precursor, and the red highlight is where the mature sequence is located.

The signature hairpin structure of miRNA precursors can be used to predict new miRNAs. As shown in Table 3, the number of miRNAs was the highest in the C4 group, 103,663, and the number of miRNAs was the least in the C1 group, 60,344. However, in the C3 group, the types of miRNAs were the most, 2,985; the C1 group had the least types, 2,318. We listed the secondary structure of the 10 predicted new miRNA on the match (Figure 10).

**TABLE 3:** Statistical table of predicted new miRNA and comparison of miRNA of each sample.

Types	Total	C1	C2	C3	C4
Mapped mature	75	65	70	72	73
Mapped star	56	38	43	48	44
Mapped hairpin	84	78	80	81	81
Mapped uniq miRNA	10,967	2,318	2,837	2,985	2,827
Mapped total miRNA	346,364	60,344	80,423	101,934	103,663

### Screening and Identification of Differential miRNAs

**FIGURE 11:**
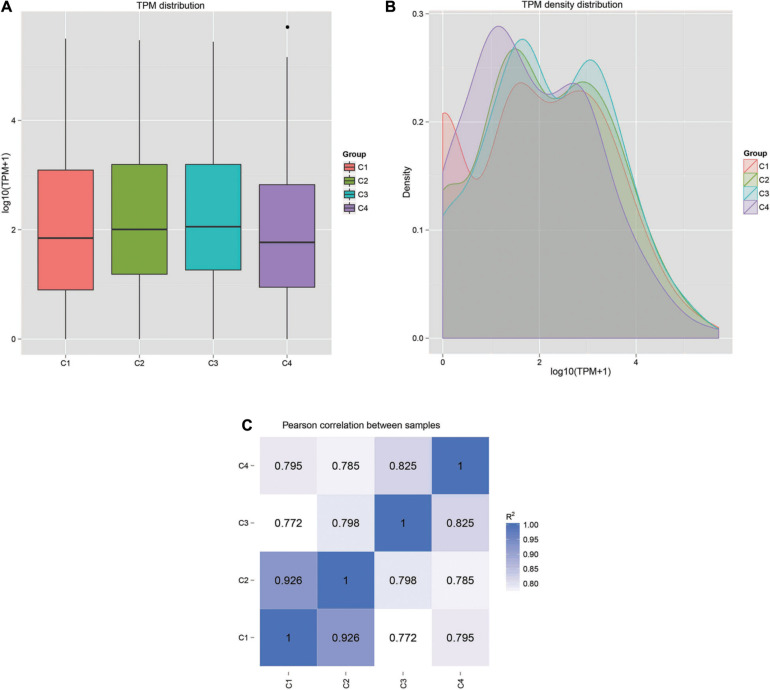
Gene expression patterns and correlations in each sample. **(A)** Box plot of TPM distribution in all groups. **(B)** TPM density distribution diagram of miRNA expression levels. **(C)** Pearson correlation of miRNA expression between samples. C1: *BcL.1-25*, C2: *BcL.1-4*, C3: *BcL.2-25*, C4: *BcL.2-4*.

**FIGURE 12:**
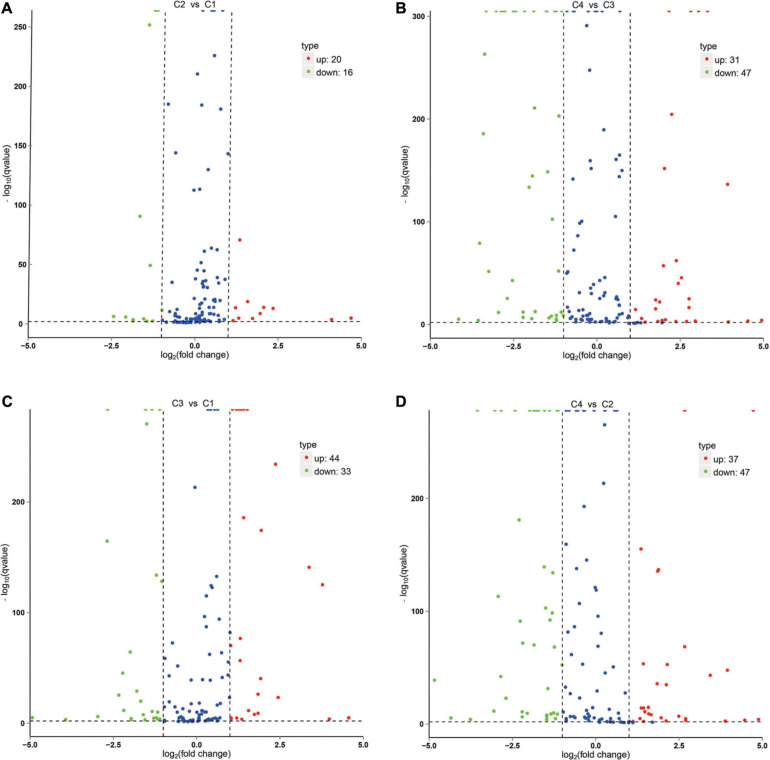
Volcano plot of differential miRNA. **(A)** Differential miRNA from C2 vs. C1. **(B)** Differential miRNA from C4 vs. C3. **(C)** Differential miRNA from C3 vs. C1. **(D)** Differential miRNA from C4 vs. C2. *X*-axis, miRNA expression fold change in different experimental groups/different control groups; *Y*-axis, statistical significance of the change in the expression level of miRNA. Red dots indicate significantly differential upregulated miRNAs, green dots indicate significantly differential downregulated miRNAs, and blue dots indicate those without significantly differential miRNAs. C1: *BcL.1-25*, C2: *BcL.1-4*, C3: *BcL.2-25*, C4: *BcL.2-4*.

The correlation analysis of gene expression levels between samples was carried out to test the reliability of the experimental results and the rationality of sample selection. In Figure 11C, R^2^, the square of the Pearson correlation coefficient, was basically at 0.772–1, indicating that the similarity of expression patterns between samples is higher. In Figure 11, the TPM density distributions of miRNA under different experimental conditions were compared. Finally, by using volcano plots, we inferred the overall distribution of differential miRNA. Differential miRNAs were screened based on fold changes in levels and corrected significance levels (padj/q value). In the C2 and C1 groups, 20 differential miRNAs were upregulated, and 16 differential miRNAs were downregulated. In the C4 and C3 groups, 31 differential miRNAs were upregulated, and 47 differential miRNAs were downregulated. In the C3 and C1 groups, 44 differential miRNAs were upregulated, and 33 differential miRNAs were downregulated. In the C4 and C2 groups, 37 differential miRNAs were upregulated and 47 differential miRNAs were downregulated (Figure 12).

### Cluster Analysis of Differential miRNAs

**FIGURE 13:**
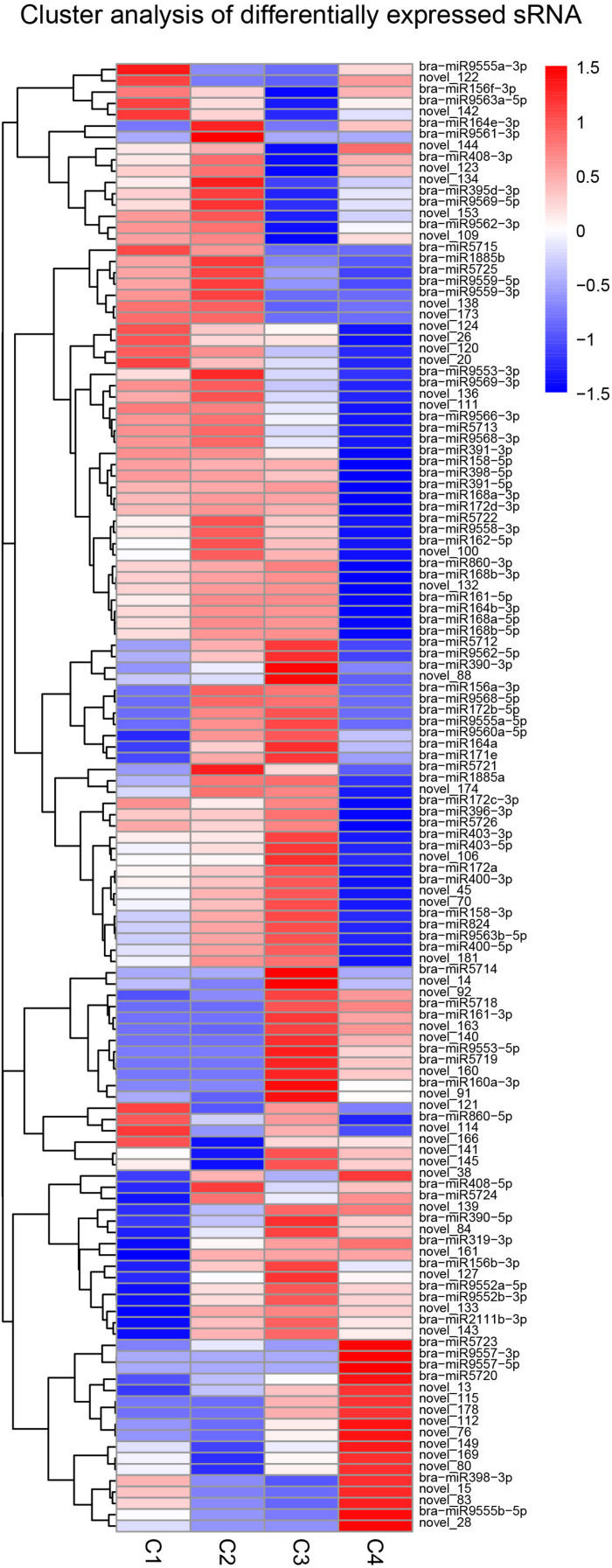
Cluster analysis of differentially expressed miRNA. The above figure is the overall hierarchical clustering chart. Clustering is performed by the log_10_ (TPM + 1) value. Red indicates high expression miRNA, and blue indicates low expression miRNA.

The clustering pattern of differential miRNA expression under different experimental conditions was determined by using differential miRNA cluster analysis. For each comparison combination, a set of differential miRNAs was obtained and was used for hierarchical clustering analysis. The number of miRNAs with high expression levels of C1, C2, and C3 was higher than that of the C4 group. In addition, the number of highly expressed miRNAs was the highest in the C2 group, while C4 had the lowest. In the C4 group, while some miRNAs had comparatively lower expression levels, others, such as bra-miR9557-3p and bra-miR9557-5p, were expressed at very high levels (Figure 13).

### Venn Diagram of Differential miRNAs

**FIGURE 14:**
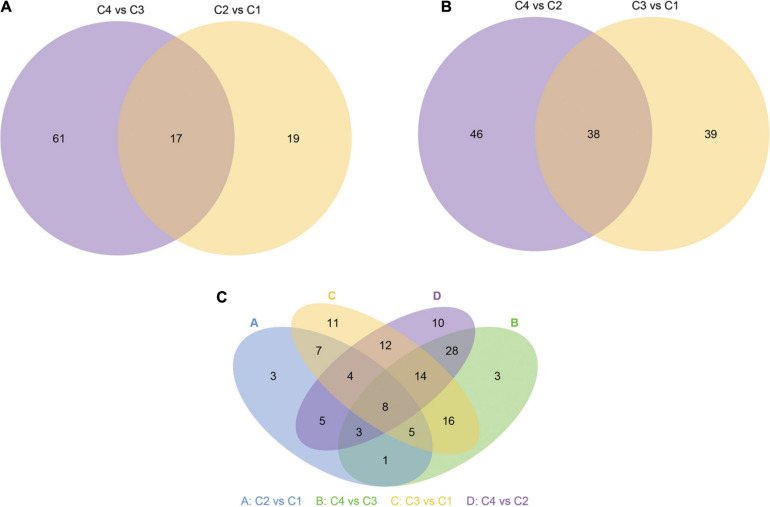
Venn diagram of differential miRNA. The large circles represent each comparison combinations, and the sum of the number in each large circle represents the total number of differential miRNAs from the comparison combinations, and the overlapping portions of the circles represent the number of common differential miRNAs between the combinations. **(A)** Venn diagram of differential miRNA between C4 vs C3 and C2 vs C1 groups. **(B)** Venn diagram of differential miRNA between C4 vs C2 and C3 vs C1 groups. **(C)** Venn diagram of differential miRNA in all groups. C1: *BcL.1-25*, C2: *BcL.1-4*, C3: *BcL.2-25*, C4: *BcL.2-4*.

Next, we show more intuitively the common and unique differences of each comparison combination. When the number of miRNAs was greater than or equal to two and less than or equal to five, the number of differential miRNAs, which was obtained by comparison in each group, can be counted and plotted as a Venn diagram (Figure 14). In Figure 14A, there were 17 common differential miRNAs; while in Figure 14B, there were 38 common differential miRNAs. In all combinations, eight common differential miRNAs are shown in Figure 14C (Table 4 and Supplementary Table 18).

**TABLE 4:** Number of combined difference (DIFF), upregulated (UP), and downregulated (DOWN) miRNAs in each group.

Group	Diff	Up	Down
C2 vs. C1	36	20	16
C4 vs. C3	78	31	47
C3 vs. C1	77	44	33
C4 vs. C2	84	37	47

### Enrichment Analysis of Differential miRNA Candidate Target Genes

After obtaining the differentially expressed miRNAs between the groups, according to the correspondence between the miRNA and its target genes, we performed GO and KEGG enrichment analysis on the set of target genes of each group of differentially expressed miRNAs.

**FIGURE 15:**
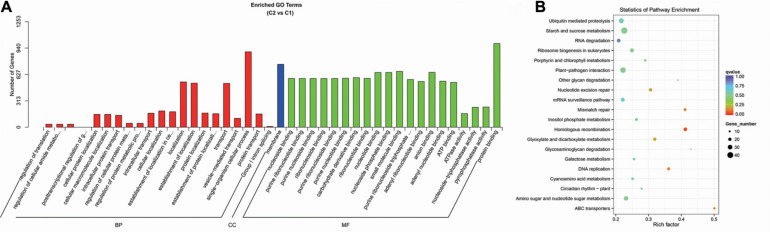
Enrichment analysis of candidate target genes for differentially expressed miRNA between the C2 and C1 groups. **(A)** Histogram of GO enrichment for candidate target genes from C2 vs. C1. Three basic classifications of Go term (from left to right, BP, biological processes; CC, cellular components; MF, molecular functions). **(B)** KEGG enrichment scatter plot of candidate target genes from C2 vs. C1. *X*-axis, the Rich factor; *Y*-axis, the name of the pathway. The size of the dots indicates the number of candidate target genes in this pathway, and the colors of the dots correspond to different Q value ranges. C1: *BcL.1-25*, C2: *BcL.1-4*, C3: *BcL.2-25*, C4: *BcL.2-4*.

Through the GO enrichment stratification analysis, between the C2 and C1 groups, single-organism cellular process (GO:0044763, 896 genes), membrane (GO:0016020, 747 genes), and protein binding (GO:0005515, 994 genes) were the most representative GO terms in biological process, cellular component, and molecular function, respectively (Figure 15A and Supplementary Table 19). Between the C4 and C3 groups, single-organism cellular process (GO:0044763, 1,748 genes), membrane (GO:0016020, 1,431 genes), and protein binding (GO:0005515, 1,973 genes) were the most representative GO terms (Supplementary Figure 4A and Supplementary Table 21). Between the C3 and C1 groups, single-organism cellular process (GO:0044763, 1,841 genes), membrane (GO:0016020, 1,511 genes), and protein binding (GO:0005515, 2,041 genes) were the most representative GO terms (Supplementary Figure 5A and Supplementary Table 23). Finally, between the C4 and C2 groups, single-organism cellular process (GO:0044763, 1,880 genes), membrane (GO:0016020, 1,515 genes), and ion binding (GO:0005515, 2,198 genes) were the most representative GO terms (Supplementary Figure 6A and Supplementary Table 25).

Through the KEGG pathway enrichment analysis, between the C2 and C1 groups, the starch and sucrose metabolism pathway was the largest, with 42 genes, followed by the plant–pathogen interaction pathway, with 36 genes (Figure 15B and Supplementary Table 20). Between the C4 and C3 groups, the starch and sucrose metabolism pathway, was the largest, with 74 genes, followed by the plant–pathogen interaction pathway, with 67 genes (Supplementary Figure 4B and Supplementary Table 22). Between the C3 and C1 groups, the plant–pathogen interaction pathway, with 68 genes, and the RNA transport pathway, also containing 68 genes, are both the pathways with the most genes (Supplementary Figure 5B and Supplementary Table 24). Between the C4 and C2 groups, the plant–pathogen interaction pathway was the largest, with 86 genes, followed by the starch and sucrose metabolism pathway, with 78 genes (Supplementary Figure 6B and Supplementary Table 26).

## Discussion

As a convenient tool for transcriptome analysis, RNA-Seq, has been increasingly targeted in species that do not have access to genomic sequences (Li et al., 2013; Liu et al., 2013; Soetaert et al., 2013). Here, we used RNA-Seq to measure the gene expression levels in non-heading Chinese cabbage of different varieties and under low-temperature treatment. To obtain all the DEGs from RNA-Seq data, the expression of all genes was analyzed depending on the RPKM. By comparing the data from each groups, we found that between the *BcL.1-25* and *BcL.1-4* groups, the most differentially expressed genes (6,208 DEGs) were enriched, and their number was almost six times that of the other groups, and the difference was the greatest. At the same time, the ribosome pathway is worth mentioning, and the most abundant genes (287 genes) were also shown, far more than those of the other groups. The results showed that cold stress could affect the genes involved in the expression of these pathways. Previous reports have also detected these rich pathways, partially reflecting the credibility of our results (Kreps et al., 2002; Pang et al., 2013). This also suggests that the ribosomal pathway might be involved in cold stress.

RNA-Seq was searched for some low temperature-related differentially expressed genes. qRT-PCR was used to identify gene expression levels (Taylor et al., 2010). To confirm the reliability of the RNA-Seq results, we performed qRT-PCR experiment to identify them. According to the qRT-PCR results, the expression patterns of all unigenes were consistent with the transcriptome sequencing data, showing that our experimental results were reliable.

Small RNAs are short, non-coding RNAs, usually 19–25 nt in length, and two protruding sizes of 21 and 24 nt, respectively (Kim et al., 2012). In general, an miRNA corresponds to a 21 nt class of small molecule RNA. Research have also found that small RNAs showed a wide range of functions, including heterochromatin formation, gene silencing, and DNA methylation (Lippman and Martienssen, 2004; Penterman et al., 2007). By small RNA sequencing, the result showed that a 24-nt length is the most abundant category, among pure and unique reads in all groups (Figure 8). Our result was highly consistent with previous studies on *A. thaliana* (Rajagopalan et al., 2006), *Oriza sativa* (Zhu et al., 2008), *Medicago truncatula* (Szittya et al., 2008), and *Populus trichocarpa* (Puzey et al., 2012).

Furthermore, the known miRNAs are analyzed, and new miRNAs are predicted. We discovered that these selected known miRNAs have at least two stem-loop structures that are obtained by self-folding (Figures 9A–J), suggesting that there might be protein or chromosomal binding sites. Meanwhile, these predicted miRNAs have at least two stem-loop structures that are obtained by self-folding (Figures 10A–J), suggesting that there might also be protein or chromosomal binding sites. The functions of these miRNAs and their target genes were comprehensively analyzed and might provide new insights into miRNA-mediated epigenetic control of Pak-choi under low temperature.

To better understand the functional roles of these predicted miRNA targets, the target genes, which were functionally annotated in biological processes, and the KEGG pathway was also used to describe the corresponding metabolic pathways. After low-temperature treatment, most target genes were enriched in the metabolic pathways of starch and sucrose, and was basically consistent with the results of the abovementioned transcriptome study. This indicated that a series of biosynthetic and metabolic pathways might be induced after low-temperature treatment (Hu et al., 2016). Here, a combination of transcriptome and small RNA sequencing was used to analyze the cold tolerance of non-heading Chinese cabbage. This study might promote further molecular regulation mechanisms of cold tolerance in Pak-choi.

## Conclusion

In this study, a total of 63.43 Gb clean data were obtained from the transcriptome analysis. Based on the comparison results, a total of 1,860 new genes were discovered in Pak-choi, and 13 common DEGs were detected in all treatments, including seven upregulated and six downregulated. Some of the DEGs were involved in response to abiotic stresses, such as freezing, cold, and salt stresses. Among them, the cold stress response is more obvious, so we used qRT-PCR experiment to confirm changes in the expression levels of these genes. Furthermore, we performed miRNA sequencing analysis on the same material. We found that the results revealed a total of 34,182,333 small RNA reads and a total of 88,604,604 kinds of small RNA, among which the most common size was 24 nt. In all materials, the number of common differential miRNAs was eight. The number of known mature miRNAs and the number of precursors were 110 and 80, respectively. The number of predicted novel miRNA matures, and the numbers of precursors were 75 and 84, respectively. According to the GO and KEGG enrichment analysis, single-organism cellular process in the biological process, membrane in the cellular component, and protein binding in the molecular function were almost all the most representative level GO terms in all data sets, while the starch and sucrose metabolism pathway was almost all the largest, followed by the plant–pathogen interaction pathway. Our findings highlighted the significance of cold signaling in Pak-choi and might provide a foundation for subsequent research under abiotic stress in the future.

## Materials and Methods

### Plant Growth Environment and Treatment Conditions

The materials used in this study were non-heading Chinese cabbage varieties, *Suzhouqing* (*BcL*.*1*) and *Sijiucaixin* (*BcL*.*2*), provided by the Nanjing Agricultural University, Cabbage System Biology Laboratory. *Suzhouqing* (*BcL*.*1*) and *Sijiucaixin* (*BcL*.*2*) were both laboratory-specific and commonly used varieties, stored in an open herbarium in the laboratory. Seedlings were transferred into plastic pots containing a mixture of soil and vermiculite (volume ratio is 3:1), and cultured in a growth chamber under 16 h light (22°C)/8 h dark (18°C). After growing for about 30 days, the plants were moved to light incubators at 4°C (*BcL*.*1-4*, *BcL*.*2-4*) and 25°C (*BcL*.*1-25*, *BcL*.*2-25*) for up to 6 h, respectively. The leaves of plants were harvested, immediately frozen in liquid nitrogen, and stored at −80°C for experimental purpose.

### Transcriptome Sequencing

Following the manufacturer’s instructions, we used RNAiso Plus reagent (TaKaRa, Dalian, China) to extract total RNA from the samples. RNA samples were examined by using a spectrophotometer and electrophoresed on a 1% agarose gel. cDNA library construction and transcriptome sequencing were performed by Biomarker Technologies (Beijing, China). Transcriptome sequencing was done by Beijing Biomarker Biotechnology^1^ Co., Ltd. By Illumina HiSeq X 10 sequencing platform and Pe150 mode sequencing, all clean reads were subsequently mapped to the *Brassica rapa* reference genome sequence (*IVFCAASv1*)^2^. The clean reads of each sample sequence were aligned with the designated reference genome. Gene expression analysis was performed based on the comparison results; differentially expressed genes were identified based on their expression levels in different samples, and their functional annotation and enrichment analysis were also performed (Wang et al., 2017).

### Transcriptome Assembly and Functional Annotation

The raw data of the transcriptome sequencing were purified by trimming adapters, removing reads containing poly-N, and rejecting the low-quality data (*quality value* ≤ 10 or unknown nucleotides larger than 5%) to get the clean reads. Meanwhile, the proportion of nucleotides with quality values greater than 30 (Q30) and GC content of the clean data were calculated. Then, all of the clean reads were assembled, using the Trinity program (Grabherr et al., 2011). First, the certain short reads with overlap regions were assembled into longer contiguous sequences for each library. Then, the distance of different contigs was recognized, mapping the clean reads, based on the paired-end information, to obtain the sequence of the transcripts. Finally, the unigenes were obtained, performing the sequence of potential transcript to the TGI Clustering tool (Pertea et al., 2003). The new genes discovered were performed with NR (Deng et al., 2006), Swiss-Prot (Apweiler et al., 2004), GO (Ashburner et al., 2000), COG (Tatusov et al., 2000), KOG (Koonin et al., 2004), Pfam (Finn et al., 2013), and KEGG (Kanehisa et al., 2004) databases, using BLAST (Altschul et al., 1997) software. KOBAS2.0 (Xie et al., 2011) was used to obtain the KEGG orthology result of the new gene for sequence alignment. After predicting the amino acid sequence of the new gene, we used the HMMER (Eddy, 1998) software to align with the Pfam database and obtained the annotation information of the new gene.

### Differentially Expressed Gene Analysis

Following fragments per kilobase of exon per million fragments mapped reads (FPKM) method, the expression level of unigene was calculated (Mortazavi et al., 2008). The ratio of the FPKM values (using 0.001 instead of 0 if the FPKM was 0) was taken as the fold changes in the expression of each gene to identify DEGs between each groups. The false discovery rate (FDR) control method was used to identify the threshold of the *p-value* in multiple tests and to compute the significance of the difference in transcript abundance (Reiner et al., 2003). In this result, only fold change with | log_2_ (case_FPKM/control_FPKM)| ≥ 1, and an FDR ≤ 0.001 were taken as the threshold for significantly differential expression. The log_2_-transformed FPKM value for DEGs was applied to generate heat map by MeV 4.7 (Howe et al., 2011). Meanwhile, the DEGs were annotated with the GO and KEGG databases.

### Validation of Differentially Expressed Genes With Quantitative Real-Time Polymerase Chain Reaction

qRT-PCR was used to confirm the expression of common differentially expressed genes in Pak-choi. Total RNA was extracted from each sample. The first-strand cDNA was synthesized, using a PrimeScript^TM^ II First Strand cDNA synthesis kit (TaKaRa Bio, Dalian, China), according to the manufacturer’s protocol. The primers were designed, using the software Beacon Designer 7.9, and listed in Table 5. The quantified expression levels of the tested genes were normalized against the housekeeping genes Cyclophilin 1 (CYP1) (Ma et al., 2016). The qRT-PCR assays were performed with three biological and technical replicates. Each reaction was performed in 20-μl reaction mixtures containing a diluted cDNA sample as template, SYBR Premix Ex Taq (2×) (TaKaRa, Kyoto, Japan) and gene-specific primers. Conditions for quantitative analysis were as follows: 95°C for 3 min, then 40 cycles (95°C 30 s, 60°C 30 s), and 72°C for 30 s. qRT-PCR was performed according to a previous report (Song et al., 2016). The comparative Ct value method was adopted to analyze the relative gene expression according to a previous analysis and RNA expression levels relative to the actin gene were calculated as 2^–ΔΔ*CT*^ (Pfaffl, 2001; Song et al., 2016).

**TABLE 5:** Primers used in the paper.

Primer name	Forward primer sequence (5′–3′)	Reverse primer sequence (5′–3′)
evm.TU.BraA01003362	AAACTTCCCAAATCTCAA	TGTAGACTCATCCTTCAT
evm.TU.BraA03001657	AGGATGTGATAAGGTAAC	ATCTCAGGTCTAACTATG
evm.TU.BraA06001385	CTAACATCATCGTTGAGTAT	CATAAGGAGTGGAAGGTA
evm.TU.BraA07000744	TGAAGGAGTGTTGGCATA	TCGTTGAGTGATGAAGAGT
evm.TU.BraA09000675	CGCCGAGAATACTACCAT	ACCGAGTGCTAAGAAGAG
evm.TU.BraA09001549	GCTTCTTCAACCATCATC	TGTCTAATCTTCTTCTTCTCT
evm.TU.BraA10002276	ATTAAGGCTTACGCAATG	CCAATGATGAGTCCAATG
Brassica_rapa_newGene_1153	GGTAATAGGCGACTGGATA	CAATGAACTGGCTCTACG
evm.TU.BraA01000616	TCTTCTCCTGATGACTGT	CTTCTTCTTCCTCCTCTTC
evm.TU.BraA06001282	ATGGCAACGAATAGTGAGA	GAGGTTACAGTAGAAGATGGT
evm.TU.BraA08001363	GGAAGACTATACTATGACAA	TAAGGAAGCAGAACAGGAA
evm.TU.BraA09000654	CGAGTTATCAGAGGCAATC	TGACGAGATGACTGTGTT
evm.TU.BraA10000511	CTTCCTAAGTTAGCCAATCT	CTGCCACAAGGTAGTTAT
qBcACTIN	GTTGCTATCCAGGCTGTTCT	AGCGTGAGGAAGAGCATAAC

### Small RNA Sequencing

By high-throughput sequencing (such as Illumina HiSeqTM2500/MiSeq and other sequencing platforms), sequenced raw image data files were converted into sequenced reads by base calling analysis. The raw data, containing the sequence information of read sequences and the corresponding sequencing quality information, were stored in FASTQ (abbreviated as fq) file format. Referring to the standard definition of miRNA (Allen et al., 2005; Schwab et al., 2005), the candidate target gene of miRNA was compared as the query sequence with the *Brassica rapa* database^3^. The control samples and inoculation samples were mixed for small RNA library construction, respectively. According to the reported procedures, the construction of small RNA libraries were completed (Sunkar et al., 2008). Three micrograms of total RNA per sample was used as input material for the small RNA library (Huang et al., 2018). Small RNA library construction and small RNA deep sequencing proceeded following the detailed protocol provided by the genome sequencing company (Novogene, China).

### Bioinformatic Analysis of Sequence Data

The raw data were first processed through custom Perl and Python scripts. The clean data were mapped to the reference sequence in miRBase21.0 by Bowtie (Langmead et al., 2009), without mismatch to look for known miRNAs. Then, the other reads were integrated to predict novel miRNAs using the available miREvo (Wen et al., 2010) and miRDeep2 (Friedländer et al., 2011) software. The miRNA counts as well as base bias were identified by using custom scripts. Then, the miFam.dat^4^ was used to look for families of known miRNAs. The novel miRNA precursor was submitted to Rfam^5^ to look for Rfam families.

### Venn Diagrams of Known miRNAs and Novel miRNAs

Normalization formula (normalized expression = mapped read count/total reads ^∗^ 1,000,000) was used to estimate miRNA expression levels (Zhou et al., 2010). DEG seq R package was used to analyze the differential expression of two samples with the criterion of Q < 0.01 and | log_2_ (fold change)| > 1 (Storey, 2003).

### Construction of Degradome Libraries

Target genes of candidate miRNAs were verified by degradome sequencing by using total RNA, the same as the RNA used for small RNA sequencing library construction, following the published parallel analysis of RNA End (PARE) protocol (German et al., 2009). The data analysis was processed, following the procedure instructions (Novogene, China).

### Target Gene Prediction and Annotation for Known and Novel miRNAs

The psRobot_tarin psRobot was performed to predict target genes of miRNA (Wu et al., 2012). To further explore the detailed molecular mechanism of miRNAs in Pak-choi response to cold stress, the target transcripts of differentially expressed miRNAs were analyzed by GO and KEGG functional annotation suites. Subsequently, the Revigo tool^6^ was implemented for enrichment analysis of the target genes. The KOBAS software was used to test the statistical enrichment of the target gene candidates in the KEGG pathways (Mao et al., 2005).

## Data Availability Statement

The full data sets have been submitted to NCBI Sequence Read Archive (SRA) under Accession SUB8670775, Bioproject: PRJNA682276.

## Author Contributions

JW completed the relevant experiments and wrote the manuscript. QZ, XY, and XH modified and approved the manuscript. XY and XH interpreted the results and coordinated the research. All authors have read and finalized the draft, agreeing that the manuscript will be submitted to this prestigious journal.

## Conflict of Interest

The authors declare that the research was conducted in the absence of any commercial or financial relationships that could be construed as a potential conflict of interest.

## Footnotes

1 http://wwwF.biomarker.com.cn/

2 http://brassicadb.org

3 http://brassicadb.org/brad/

4 http://www.mirbase.org/ftp.shtml

5 http://rfam.sanger.ac.uk/search/

6 http://revigo.irb.hr

## Supplementary Material

Supplementary Figure 1 Classification diagram of KEGG pathway types. **(A)** KEGG pathway types from C2 vs. C1. **(B)** KEGG pathway types from C4 vs. C3. **(C)** KEGG pathway types from C3 vs. C1. **(D)** KEGG pathway types from C4 vs. C2. *X*-axis, the number of genes in this pathway and their ratio to the total number of genes; *Y*-axis, the name of the KEGG metabolic pathway. C1: *BcL.1-25*, C2: *BcL.1-4*, C3: *BcL.2-25*, C4: *BcL.2-4*.

Supplementary Figure 2 COG classification of differentially expressed genes. **(A)** COG classification from C2 vs. C1. **(B)** COG classification from C4 vs. C3. **(C)** COG classification from C3 vs. C1. **(D)** COG classification from C4 vs. C2. *X*-axis, the specific content of each COG classification, *Y*-axis, the number of genes. C1: *BcL.1-25*, C2: *BcL.1-4*, C3: *BcL.2-25*, C4: *BcL.2-4*.

Supplementary Figure 3 Cluster analysis of differentially expressed genes. *X*-axis, name and clustering results of samples; *Y*-axis, differential gene and gene clustering results. Different columns in the figure represent different samples, and different rows represent different genes. The color represents the expression level log_10_ (FPKM + 0.000001) of the gene in the sample.

Supplementary Figure 4 Enrichment analysis of candidate target genes for differentially expressed miRNA between C4 and C3 groups. **(A)** Histogram of GO enrichment for candidate target genes from C4 vs. C3. Three basic classifications of Go term (from left to right, BP: biological processes, CC: cellular components, MF: molecular functions). **(B)** KEGG enrichment scatter plot of candidate target genes from C4 vs. C3. *X*-axis, the Rich factor; *Y*-axis, the name of the pathway. The size of the dots indicates the number of candidate target genes in this pathway, and the colors of the dots correspond to different Q-value ranges. C1: *BcL.1-25*, C2: *BcL.1-4*, C3: *BcL.2-25*, C4: *BcL.2-4*.

Supplementary Figure 5 Enrichment analysis of candidate target genes for differentially expressed miRNA between C3 and C1 groups. **(A)** Histogram of GO enrichment for candidate target genes from C3 vs. C1. Three basic classifications of Go term (from left to right, BP: biological processes, CC: cellular components, MF: molecular functions). **(B)** KEGG enrichment scatter plot of candidate target genes from C3 vs. C1. *X*-axis, the Rich factor; *Y*-axis, the name of the pathway. The size of the dots indicates the number of candidate target genes in this pathway, and the colors of the dots correspond to different Q-value ranges. C1: *BcL.1-25*, C2: *BcL.1-4*, C3: *BcL.2-25*, C4: *BcL.2-4*.

Supplementary Figure 6 Enrichment analysis of candidate target genes for differentially expressed miRNA between C4 and C2 groups. **(A)** Histogram of GO enrichment for candidate target genes from C4 vs. C2. Three basic classifications of Go term (from left to right, BP: biological processes, CC: cellular components, MF: molecular functions). **(B)** KEGG enrichment scatter plot of candidate target genes from C4 vs. C2. *X*-axis, the Rich factor; *Y*-axis, the name of the pathway. The size of the dots indicates the number of candidate target genes in this pathway, and the colors of the dots correspond to different *Q*-value ranges. C1: *BcL.1-25*, C2: *BcL.1-4*, C3: *BcL.2-25*, C4: *BcL.2-4*.

Supplementary Table 1 Thirteen common differentially expressed genes (seven up-regulated and six down-regulated) in all materials.

Supplementary Table 2 GO enrichment analysis of differentially expressed genes between C2 and C1 groups.

Supplementary Table 3 KEGG enrichment analysis of differentially expressed genes between C2 and C1 groups.

Supplementary Table 4 GO enrichment analysis of differentially expressed genes between C4 and C3 groups.

Supplementary Table 5 KEGG enrichment analysis of differentially expressed genes between C4 and C3 groups.

Supplementary Table 6 GO enrichment analysis of differentially expressed genes between C3 and C1 groups.

Supplementary Table 7 KEGG enrichment analysis of differentially expressed genes between C3 and C1 groups.

Supplementary Table 8 GO enrichment analysis of differentially expressed genes between C4 and C2 groups.

Supplementary Table 9 KEGG enrichment analysis of differentially expressed genes between C4 and C2 groups.

Supplementary Table 10 KEGG enrichment pathway type of differentially expressed genes between C2 and C1 groups.

Supplementary Table 11 KEGG enrichment pathway type of differentially expressed genes between C4 and C3 groups.

Supplementary Table 12 KEGG enrichment pathway type of differentially expressed genes between C3 and C1 groups.

Supplementary Table 13 KEGG enrichment pathway type of differentially expressed genes between C4 and C2 groups.

Supplementary Table 14 COG classification statistics of differentially expressed genes between C2 and C1 groups.

Supplementary Table 15 COG classification statistics of differentially expressed genes between C4 and C3 groups.

Supplementary Table 16 COG classification statistics of differentially expressed genes between C3 and C1 groups.

Supplementary Table 17 COG classification statistics of differentially expressed genes between C4 and C2 groups.

Supplementary Table 18 Number of common differential miRNAs.

Supplementary Table 19 GO enrichment analysis of differential miRNA target genes between C2 and C1 groups.

Supplementary Table 20 KEGG enrichment analysis of differential miRNA target genes between C2 and C1 groups.

Supplementary Table 21 GO enrichment analysis of differential miRNA target genes between C4 and C3 groups.

Supplementary Table 22 KEGG enrichment analysis of differential miRNA target genes between C4 and C3 groups.

Supplementary Table 23 GO enrichment analysis of differential miRNA target genes between C3 and C1 groups.

Supplementary Table 24 KEGG enrichment analysis of differential miRNA target genes between C3 and C1 groups.

Supplementary Table 25 GO enrichment analysis of differential miRNA target genes between C4 and C2 groups.

Supplementary Table 26 KEGG enrichment analysis of differential miRNA target genes between C4 and C2 groups.

## Abbreviations

1COG: Cluster of Orthologus Groups
CYP1: Cyclophilin 1
DEGs: Differentially expressed genes
FC: Fold change
FDR: False discovery rate
GO: Gene ontology
hc-siRNA: heterochromatic short interfering RNA
KEGG: Kyoto Encyclopedia of Genes and Genomes
miRNA: microRNA
nt: nucleotides
qRT-PCR: quantitative real-time polymerase chain reaction
RNA-Seq: RNA sequencing
ta-siRNA: trans short interfering RNA.
