# Supplementary figures and images for: Transcriptome and Small RNA Combined Sequencing Analysis of Cold Tolerance in Non-heading Chinese Cabbage
Source: Front Genet. 2021 Jul 21;12:605292. doi: 10.3389/fgene.2021.605292 (PMC8334874; doi:10.3389/fgene.2021.605292)

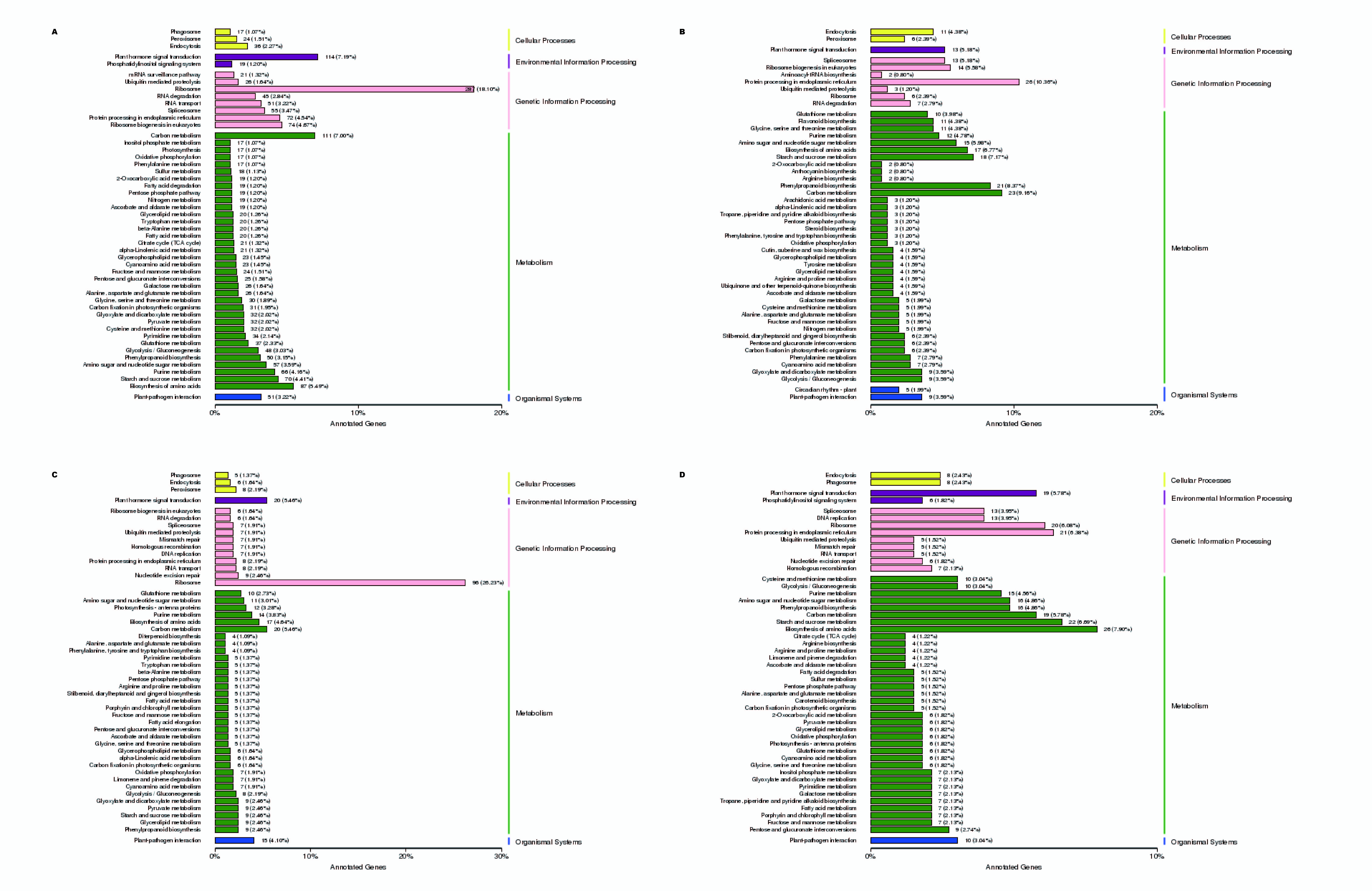

Supplement: Supplementary Figure 1 — Classification diagram of KEGG pathway types. (A) KEGG pathway types from C2 vs. C1. (B) KEGG pathway types from C4 vs. C3. (C) KEGG pathway types from C3 vs. C1. (D) KEGG pathway types from C4 vs. C2. X-axis, the number of genes in this pathway and their ratio to the total number of genes; Y-axis, the name of the KEGG metabolic pathway. C1: BcL.1-25, C2: BcL.1-4, C3: BcL.2-25, C4: BcL.2-4. [file Data_Sheet_1.zip › 2021-04-30ú1⁄4Additional files, Article Manuscript ID 605292, Proof/FIGURE S1.jpg]

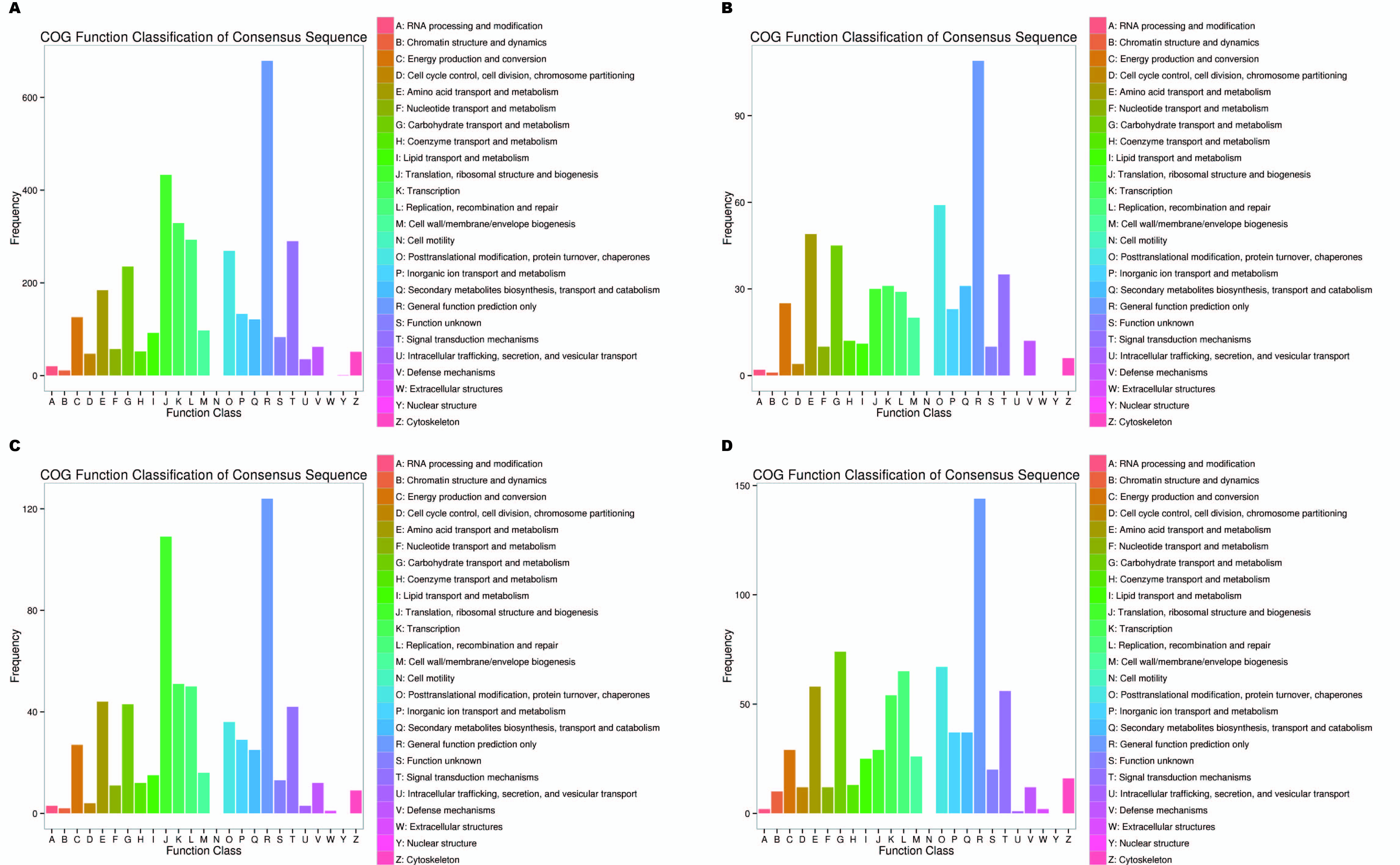

Supplement: Supplementary Figure 1 — Classification diagram of KEGG pathway types. (A) KEGG pathway types from C2 vs. C1. (B) KEGG pathway types from C4 vs. C3. (C) KEGG pathway types from C3 vs. C1. (D) KEGG pathway types from C4 vs. C2. X-axis, the number of genes in this pathway and their ratio to the total number of genes; Y-axis, the name of the KEGG metabolic pathway. C1: BcL.1-25, C2: BcL.1-4, C3: BcL.2-25, C4: BcL.2-4. [file Data_Sheet_1.zip › 2021-04-30ú1⁄4Additional files, Article Manuscript ID 605292, Proof/FIGURE S2.jpg]

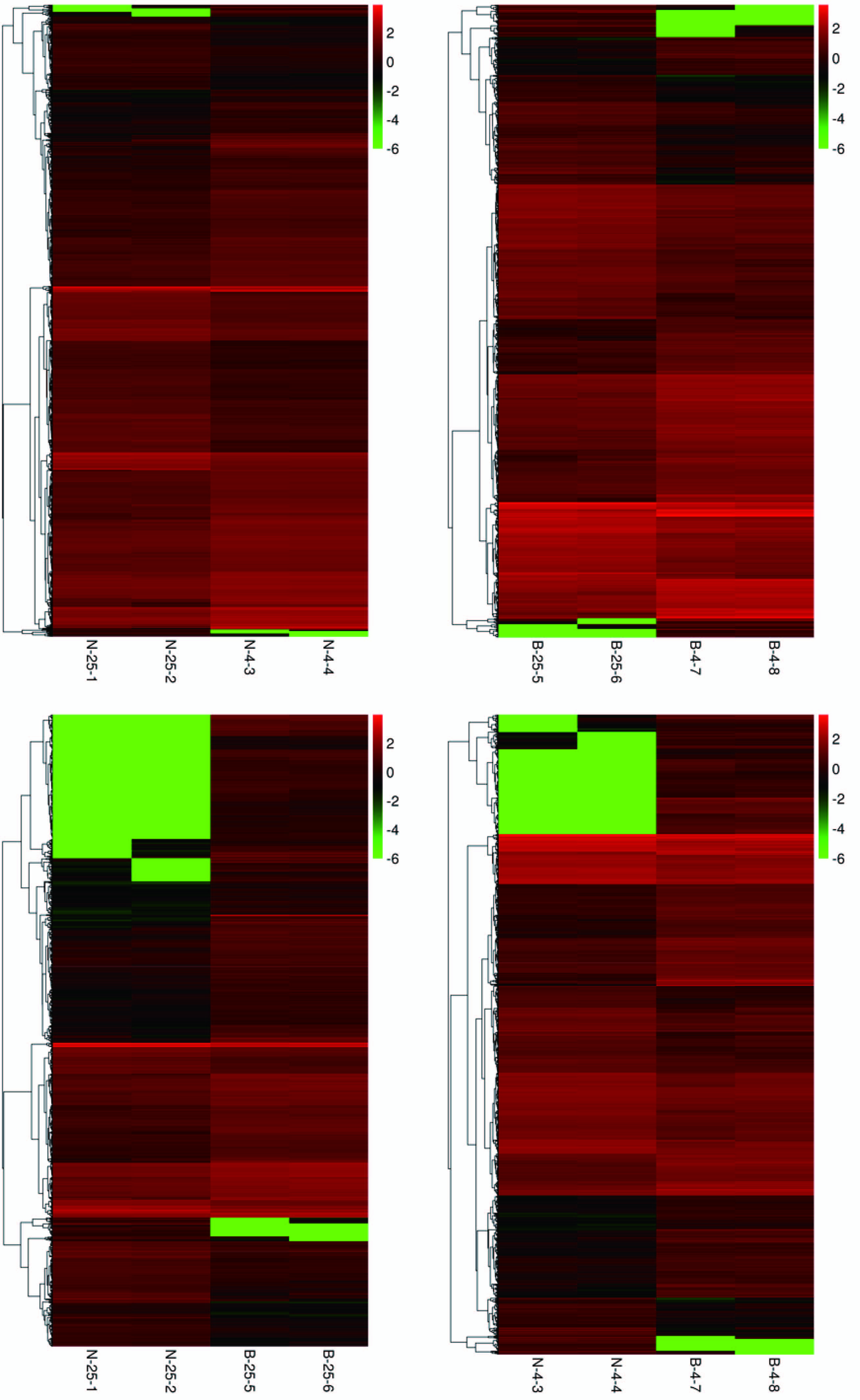

Supplement: Supplementary Figure 1 — Classification diagram of KEGG pathway types. (A) KEGG pathway types from C2 vs. C1. (B) KEGG pathway types from C4 vs. C3. (C) KEGG pathway types from C3 vs. C1. (D) KEGG pathway types from C4 vs. C2. X-axis, the number of genes in this pathway and their ratio to the total number of genes; Y-axis, the name of the KEGG metabolic pathway. C1: BcL.1-25, C2: BcL.1-4, C3: BcL.2-25, C4: BcL.2-4. [file Data_Sheet_1.zip › 2021-04-30ú1⁄4Additional files, Article Manuscript ID 605292, Proof/FIGURE S3.jpg]

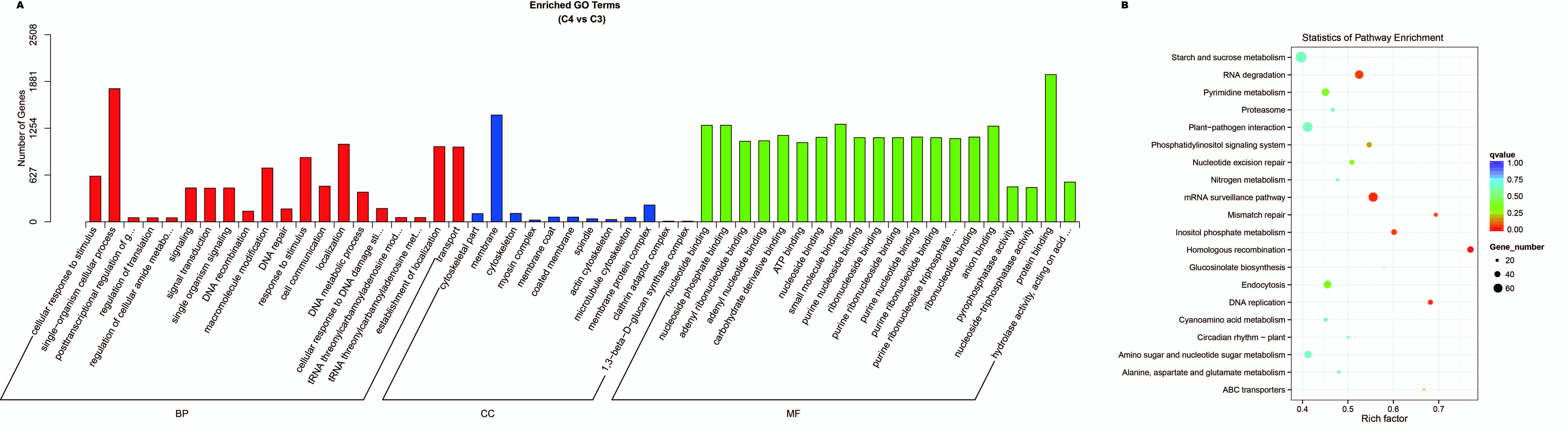

Supplement: Supplementary Figure 1 — Classification diagram of KEGG pathway types. (A) KEGG pathway types from C2 vs. C1. (B) KEGG pathway types from C4 vs. C3. (C) KEGG pathway types from C3 vs. C1. (D) KEGG pathway types from C4 vs. C2. X-axis, the number of genes in this pathway and their ratio to the total number of genes; Y-axis, the name of the KEGG metabolic pathway. C1: BcL.1-25, C2: BcL.1-4, C3: BcL.2-25, C4: BcL.2-4. [file Data_Sheet_1.zip › 2021-04-30ú1⁄4Additional files, Article Manuscript ID 605292, Proof/FIGURE S4.jpg]

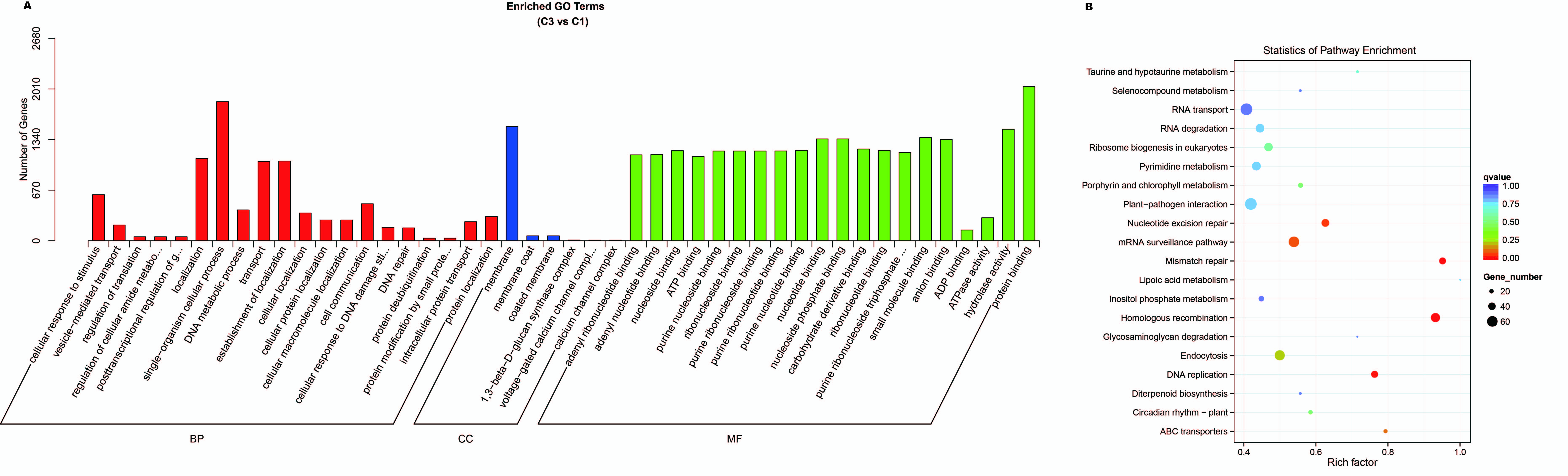

Supplement: Supplementary Figure 1 — Classification diagram of KEGG pathway types. (A) KEGG pathway types from C2 vs. C1. (B) KEGG pathway types from C4 vs. C3. (C) KEGG pathway types from C3 vs. C1. (D) KEGG pathway types from C4 vs. C2. X-axis, the number of genes in this pathway and their ratio to the total number of genes; Y-axis, the name of the KEGG metabolic pathway. C1: BcL.1-25, C2: BcL.1-4, C3: BcL.2-25, C4: BcL.2-4. [file Data_Sheet_1.zip › 2021-04-30ú1⁄4Additional files, Article Manuscript ID 605292, Proof/FIGURE S5.jpg]

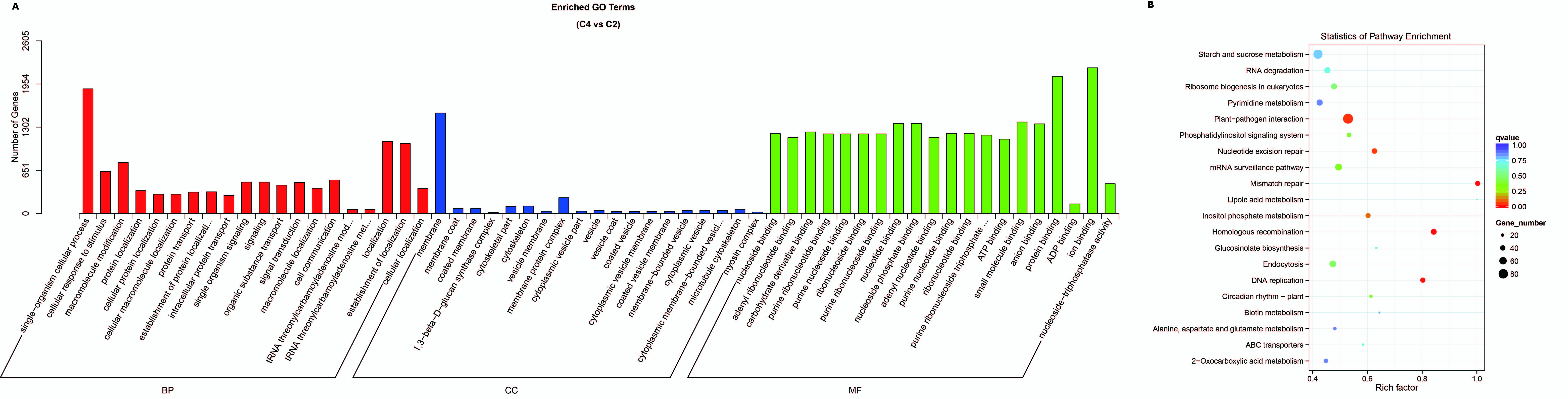

Supplement: Supplementary Figure 1 — Classification diagram of KEGG pathway types. (A) KEGG pathway types from C2 vs. C1. (B) KEGG pathway types from C4 vs. C3. (C) KEGG pathway types from C3 vs. C1. (D) KEGG pathway types from C4 vs. C2. X-axis, the number of genes in this pathway and their ratio to the total number of genes; Y-axis, the name of the KEGG metabolic pathway. C1: BcL.1-25, C2: BcL.1-4, C3: BcL.2-25, C4: BcL.2-4. [file Data_Sheet_1.zip › 2021-04-30ú1⁄4Additional files, Article Manuscript ID 605292, Proof/FIGURE S6.jpg]

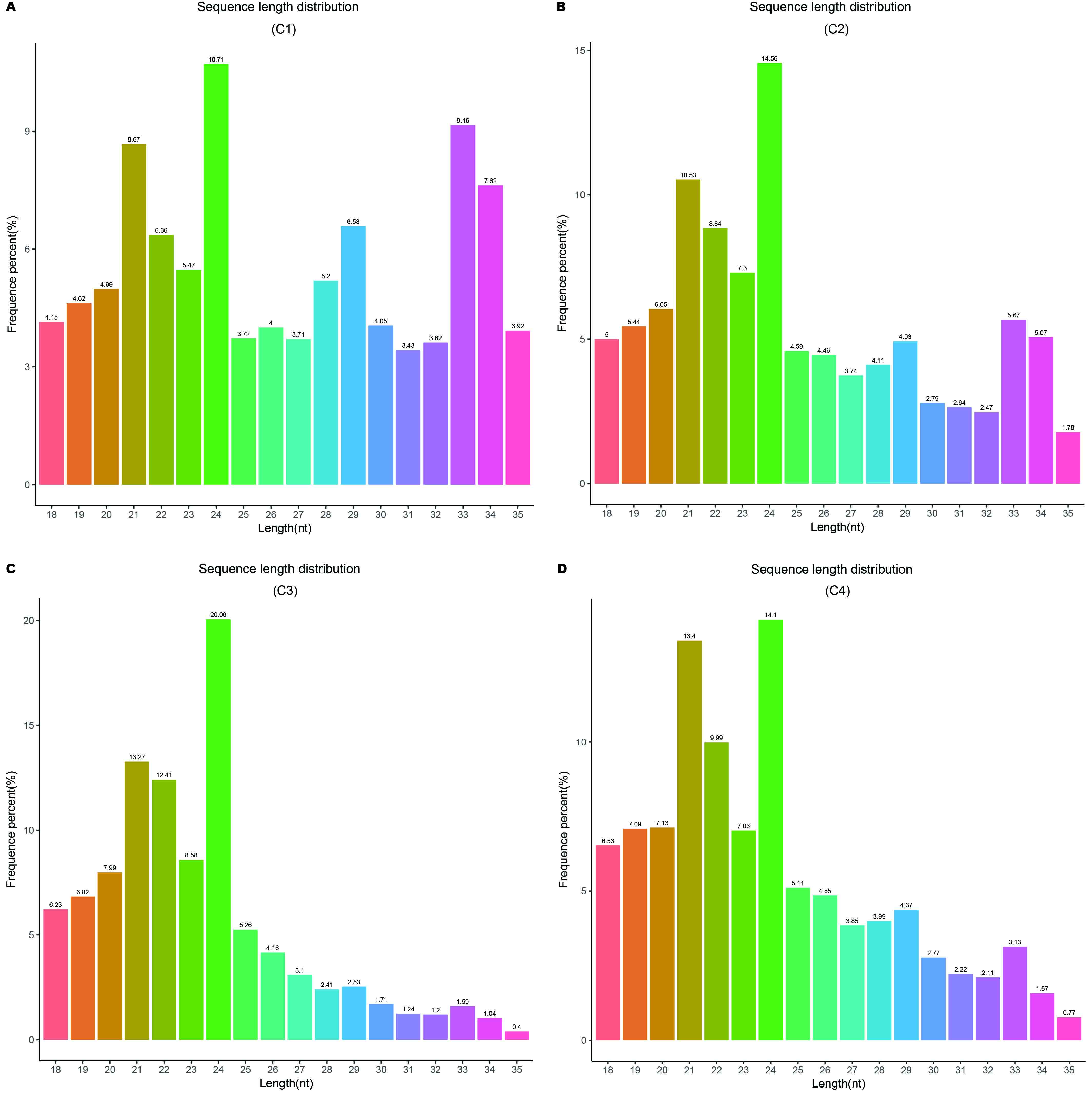

Supplement: Supplementary file 2 [file Figure_7.JPEG]
